# Supplementary material for: Perceptions on respectful maternity care in Sri Lanka: Study protocol for a mixed-methods study of patients and providers
Source: PLoS One. 2021 May 5;16(5):e0250920. doi: 10.1371/journal.pone.0250920 (PMC8099093; doi:10.1371/journal.pone.0250920)
Supplement: S1 File — (PDF) [file pone.0250920.s001.pdf]

Study title- Perceptions on respectful maternity care in Sri Lanka.

Interviewer guide for in-depth interviews of postnatal mothers who have recently delivered at Castle Street Hospital for Women and De Soysa Hospital for Women.

[START TAPE RECORDER NOW]

My name is _____________.

I am facilitating this in-depth interview on Perceptions on respectful maternity care in Sri Lanka on ___________(DATE)

at________________(WARD NUMBER) in ____________(HOSPITAL NAME).

Thank you for giving me permission to record this discussion.

We are conducting a study to help us understand your experiences in this hospital when you admitted to deliver your baby here. We are also interested in what you think may have contributed to what you experienced and what can be done to improve your experience during delivery in this hospital. In addition, we want to know your expectations of the care you think you should be receiving in this hospital.

(Always remember to pause for mothers to think and answer. Don't rush to the next question if they are still thinking about the question. It is fine to have some silence while people think. Use the probes as appropriate. Don't just accept any yes or no answer. Always probe: Why do you say that? How did that make you feel? Etc.)

Dear new mothers, have a nice day!
Could you please give me honest answers for the following questions?

Introduction

Use opening question to establish rapport and then go to the questions on basic details E.g., Where do you come from (or something more appropriate)?.

1. Is it fine if I call you 'amma'?
2. How old are you?
3. Which pregnancy is this?
4. What was the method you delivered your baby? Is it a normal or an instrumental?
5. How many weeks of pregnancy when you had your delivery?

Main component	Main questions	Probing questions	Notes
1. Birth experience	How do you feel about your birth experience?<br><br>How did you decide to deliver in this hospital?	Are you satisfied with your labour and birth?<br><br>Describe your feelings about labour and childbirth?<br><br>- Has it happened as you expected?<br>- Are you happy or tired or feeling down?<br>- Are those positive/negative memories<br>- Is it painful to remind it?<br>- Did they call by your name? How was your feeling about that?	
2. Birthing position	What do you think about your birthing position?	Are you satisfied with your birthing position, if yes or if no- explain why do you say that?<br><br>Please let me know your opinion about moving up and down during labour and childbirth including alternative positions such as sitting or squatting?	

3. Pain relief in labour	What do you think about your pain relief in labour?	Are you satisfied with pain relief in labour, if no- explain why?<br><br>Could you please describe your role in choosing a pain relief method?<br><br>Do you think that you were left with more pain during birth?	
4. Abuse-physical/sexual/verbal	What do you think about any physical violence during childbirth and labour including use of force or physical restraint?<br><br>What do you think about sexual abuse or rape in the labour ward?<br><br>What is your experience about harsh language during labour and childbirth?	Do you have any experience regarding abuse in the form of hitting or unnecessary restraints during childbirth?<br><br>Do you have any experience of exposure of your sexual parts unnecessarily or rough vaginal examinations and/or other abusive treatment?<br><br>Please describe your personal experience regarding this.<br><br>Were you subjected to harsh, rude or judgmental language?	
5. Failure to meet professional standards of care	Please describe your experience about the informed choice during labour and childbirth.<br><br>Please describe about your autonomy during labour and childbirth.	Please describe your experience about vaginal examinations?<br><br>Please describe your experience about procedures and informed consent with regards to instrumental and caesarean delivery at least?<br><br>Please kindly explain if you have any experience about humiliation or excessive pain during childbirth.	

	Please describe your experience about physical examinations and procedures?	Do you think that you were well-treated or mal-treated?<br><br>Do you think that doctors, nurses and midwives treated you with respect?<br><br>Were there any hospital staff category treat people differently? If yes-please explain why do you say so?<br><br>Did you experience any form of withholding food and drink, limited mobility, or generally a lack of kindness during your labour and childbirth?<br><br>Did you feel neglected and left alone during your labour and childbirth, if yes kindly explain why?<br><br>How did you feel about the way your baby was cared for?	
6. Labour companionship and support	Explain your experience about continuous support during labor and childbirth for the staff?	Was a female relative allowed to stay with you during your childbirth?<br><br>- If yes, how did you feel that experience?<br><br>- If no, how did you feel about that?	
7. Health system conditions and constraints	What do you feel about the adequacy of resources and policies to provide healthcare for you?<br><br>What is your honest opinion about the overall quality of healthcare you received?	Please describe your understanding about staff shortage or resource shortage?<br><br>Do you think that doctors, nurses and midwives did everything they could help you?<br><br>Could you trust them and their skills?<br><br>How was the labour room environment?	

8. Stigma and discrimination	What about your experience about stigma and discrimination during childbirth?	Did you feel any discrimination based on sociodemographic characteristics (age, income, social class, educational level)?<br><br>Did you feel any discrimination based on your ethnicity or religion?<br><br>Anybody treated you differently?	
9. Accountability	Please kindly explain your potential capacity to react for redress following violations during birth?	What do you think about your potential capacity to react in a possible violation during your childbirth?<br><br>Do you think a pathway to complain about these violation by the health staff as an essential step?	
Is there anything else you think we should know?<br><br>Do you have any questions for us?		[STOP TAPE RECORDER]	

What was it like to participate in this interview?<br><br>This is the end of our discussion. Thank you so much for taking the time to answer our questions.			
