# Supplementary material for: Perceptions on respectful maternity care in Sri Lanka: Study protocol for a mixed-methods study of patients and providers
Source: PLoS One. 2021 May 5;16(5):e0250920. doi: 10.1371/journal.pone.0250920 (PMC8099093; doi:10.1371/journal.pone.0250920)
Supplement: S2 File — (PDF) [file pone.0250920.s002.pdf]

Study title- Perceptions on respectful maternity care in Sri Lanka.

<u>Interviewer guide for in-depth interviews of healthcare providers [Doctors/Nurses/Midwives] working at Castle Street Hospital for Women and De Soysa Hospital for Women.</u>

[START TAPE RECORDER NOW]

I am a _____________. [PROFESSION- Doctor or Nurse or Midwife]

I am facilitating this in-depth interview on Perceptions on respectful maternity care in Sri Lanka on ___________(DATE)

at_____________ ____________(HOSPITAL NAME).

Thank you for giving me permission to record this discussion.

We are conducting a study to help us understand your views and perceptions about the respectful maternity care for the mothers delivering in this hospital. We are also interested in what you think may have contributed to what you perceived and what can be done and your opinion to improve respectful care for the mothers delivering in this hospital. In addition, we want to know your suggestions to improve maternal healthcare in this hospital.

(Always remember to pause for people to think and answer. Don't rush to the next question if they are still thinking about the question. It is fine to have some silence while people think. Use the probes as appropriate. Don't just accept any yes or no answer. Always probe: Why do you say that? How did that make you feel? Etc.)

Dear colleagues and staff members,
Have you a nice day!

Could you please kindly answer the following questions?

Introduction

Use opening question to establish rapport and then go to the questions on basic details E.g., Where do you come from (or something more appropriate)?.

1. Is it fine if I call you by your name?
2. How old are you?
3. What is your profession? Doctor/Midwife/Nurse
4. How many years of experience you have?

Main component	Main questions	Probing questions	Notes
1. Birth experience	Kindly explain what do you understand by a positive childbirth experience?	How do you promote a positive childbirth experience?<br><br>Please explain how can you describe the disadvantages of negative childbirth experience?<br><br>What do you understand by respectful maternity care<br><br>Have you heard of it?	
2. Mode of delivery	What is your opinion regarding taking the mother too in decision making about mode of delivery?	Please describe your personal opinion regarding normal birth?<br><br>Please describe your personal opinion regarding caesarean birth?	

		Please describe how you are supposed to educate women during antenatal period to choose a mode of delivery.	
3. Birthing position	What do you think about the birthing position in childbirth and labour?	Please describe your personal opinion regarding birthing position in childbirth and labour.<br><br>What is your honest opinion about moving up and down during labour and childbirth including alternative positions such as sitting or squatting?<br><br>Have ever allowed those alternative positions other than dorsal position?	
4. Pain relief in labour	Please let me know, what do you think about pain relief in labour?	Please describe how you choose a pain relief method.<br><br>Please describe the methods you have used during your carrier?<br><br>What are the barriers and challenges you have experienced in offering a proper pain relief method?	

5. Abuse-Physical/sexual/verbal	What do you think about any physical violence during childbirth and labour including use of force or physical restraint?<br><br>What do you think about sexual abuse or rape during pregnancy, labour and childbirth?<br><br>What is your experience about harsh language during labour and childbirth?	Have you ever done any experience regarding abuse in the form of hitting or unnecessary restraints during labour and childbirth?<br><br>Have you ever seen any experience regarding abuse in the form of hitting or unnecessary restraints during labour and childbirth?<br><br>When a mother is close to delivery or during an instrumental delivery have you ever used/seen any form of physical violence?<br><br>Have you ever seen unnecessary exposure of women's sexual parts or rough vaginal examinations and/or other abusive treatment by your colleagues?<br><br>Have you ever done harsh, rude or judgmental language to pregnant/ laboring women?	

		Have you ever seen harsh, rude or judgmental language to pregnant/ laboring women?<br><br>When a mother is close to delivery or during an instrumental delivery have you ever used/seen any form of physical violence?	
6. Failure to meet professional standards of care	Please describe your experience about offering the informed choice during pregnancy needs, childbirth and labour.<br><br>Please describe about offering autonomy during your pregnancy, childbirth and labour.<br><br>Please describe your experience about physical examinations and procedures for pregnant women?	Please describe your experience about painful vaginal examinations by yourself or your colleagues?<br><br>Please describe your experience about your role in helping women in deciding mode of delivery and considering their informed consent.<br><br>Have you ever mal-treated a labouring woman?<br><br>Have you seen any form of a lack of kindness for labouring women by the health staff?	

7. Stigma and discrimination	What about your experience about stigma and discrimination during childbirth?	Have you experienced any discrimination based on social factors (income, social class, educational level) of labouring women?<br><br>Have you treated you a mother differently?	
8. Social support	What is your kind opinion about a labour companion?	Have you ever promoted regarding the opportunity of keeping a female relative during their birth?	
9. Accountability	Your personal opinion on the women's feasibility to complain about a redress following violations during their childbirth?	What do you think about feasibility to react in a possible redress violation during your labour and childbirth at your hospital?<br><br>Do you think a mechanism to complain about these violations made by the health staff as an essential step to maintain the quality of care?	

Is there anything else you think we should know?<br><br>Do you have any questions for us?<br><br>What was it like to participate in this interview?<br><br>This is the end of our discussion. Thank you so much for taking the time to answer our questions.		[STOP TAPE RECORDER]	
