# Supplementary material for: Perceptions on respectful maternity care in Sri Lanka: Study protocol for a mixed-methods study of patients and providers
Source: PLoS One. 2021 May 5;16(5):e0250920. doi: 10.1371/journal.pone.0250920 (PMC8099093; doi:10.1371/journal.pone.0250920)
Supplement: S3 File — (PDF) [file pone.0250920.s003.pdf]

Study on perception on respectful maternity care in Sri Lanka.

Underline the correct answer please. Serial no-

Good morning, could you please kindly answer my short questions.

Part 1: Basic demographic and clinical characteristics

1. **Age in years:** 3. **Gestational age:**
2. **Parity:**
4. **Educational level:** Up to grade 5 (no formal education), Up to O/L, Up to A/L, Diploma or University degree, Postgraduate degree.
5. **Monthly Income (LKR):** <20,000, 20,000-50,000, 50,000-80,000, 80,000<.
6. **Any previous birth experiences**: vaginal, assisted vaginal, planned caesarean and emergency caesarean.
7. **Preferred [expected] mode of delivery:** vaginal or caesarean.

Part 2: Pregnant mothers' expectations for childbirth and care

1. Labour and birth went as I had expected.
   a. Extremely agree b. Agree c. Not special
   b. Disagree e. Extremely disagree

2. I felt strong during labour and birth.
   a. Extremely agree b. Agree c. Not special
   b. Disagree e. Extremely disagree

3. I felt scared during labour and birth.
   a. Extremely agree b. Agree c. Not special
   b. Disagree e. Extremely disagree

4. I felt capable during labour and birth.
   a. Extremely agree b. Agree c. Not special
   b. Disagree e. Extremely disagree

5. I was tired during labour and birth.

a. Extremely agree b. Agree c. Not special
   c. Disagree e. Extremely disagree

6. I felt happy during labour and birth.

a. Extremely agree b. Agree c. Not special
b. Disagree e. Extremely disagree

7. I have many positive memories from childbirth.

a. Extremely agree b. Agree c. Not special
d. Disagree e. Extremely disagree

8. I have many negative memories from childbirth.

a. Extremely agree b. Agree c. Not special
b. Disagree e. Extremely disagree

9. Some of my memories from childbirth make me feel depressed.

a. Extremely agree b. Agree c. Not special
e. Disagree e. Extremely disagree

10. I felt very well cared for by labour room staff.

a. Extremely agree b. Agree c. Not special
b. Disagree e. Extremely disagree

11. I felt that my baby was very well cared by labour room staff.

a. Extremely agree b. Agree c. Not special
b. Disagree e. Extremely disagree

12. Labour room environment and staff were so abusive.

a. Extremely agree b. Agree c. Not special
f. Disagree e. Extremely disagree

13. Mother's participation is mandatory in deciding the mode of delivery (vaginal or caesarean).

a. Extremely agree b. Agree c. Not special
b. Disagree e. Extremely disagree

14. I would like to come to this hospital for my next birth too.

a. Extremely agree b. Agree c. Not special
b. Disagree e. Extremely disagree

15. I would like to have a vaginal delivery for my next pregnancy too.

a. Extremely agree b. Agree c. Not special
b. Disagree e. Extremely disagree

16. As a whole, how painful did you feel childbirth was?

No pain — Worst imaginable pain

0 1 2 3 4 5 6 7 8 9 10

17. As a whole, how much control did you feel you had during childbirth?

No control — Complete control

0 1 2 3 4 5 6 7 8 9 10

18. As a whole, how secure did you feel during childbirth?

Not at all secure — Completely secure

0 1 2 3 4 5 6 7 8 9 10

Thank you for your participation!
