# Supplementary material for: Perceptions on respectful maternity care in Sri Lanka: Study protocol for a mixed-methods study of patients and providers
Source: PLoS One. 2021 May 5;16(5):e0250920. doi: 10.1371/journal.pone.0250920 (PMC8099093; doi:10.1371/journal.pone.0250920)
Supplement: S5 File — (PDF) [file pone.0250920.s005.pdf]

කාසල් වීදියේ කාන්තා රෝහල (ශික්ෂණ)
காசல் வீதி மகளிர் மருத்துவமனை (போதனா)
Castle Street Hospital for Women (Teaching)
කාසල් වීදිය, කොළඹ 8. காசல் வீதி, கொழும்பு 8. Castle Street, Colombo 8, Sri Lanka.

CERTIFICATE OF ETHICAL CLEARANCE

Investigators ; Dr.Malitha Patabendige

Application No; ERC/256/05/2019
Approval Date; 29/01/2020
Approval Expiry Date; 29/01/2021

Research Title; Maternal Perceptions and Healthcare Providers' Perceptions on Quality of Care during Labour and Childbirth: A Mixed-Methods Study

Your research proposal has been reviewed by the Ethics Review Committee of the Castle Street Hospital for Women at its meeting on 29/01/2020 and the Committee has decided to grant you approval to carry out your research in the Castle Street Hospital.

Transfer of Biological Material Overseas Yes/ No
Conflict of Interest Declared Yes/ No
Collaborators Local Yes/ No Foreign Yes/ No
Funding Local Yes/ No Foreign Yes/ No, if yes specify...........

Documents Approved

Document	Version No	Date
01.ProjectProtocol	V 1.0	31.10.2019
02.Information sheet & consent form-English	V 1.0	31.10.2019
03.Information sheet & consent form-Sinhala	V 1.0	31.10.2019
04.Information sheet & consent form-Tamil	V 1.0	31.10.2019
05.Study instrument English	V 1.0	31.10.2019
06.Study instrument-Sinhala	V 1.0	31.10.2019
07.Study instrument-Tamil	V 1.0	31.10.2019

Special Instructions;

Adverse Effects or Unforeseen; You should notify ethical review committee (ERC) immediately of any serious or unexpected adverse effect on participants of unforeseen events affecting the ethical acceptability of the project. Suspend or modify the project if the risks to participants are found to be disproportionate to the benefits. Stop any involvement of any participant if continuation of the research may be harmful to that person.

Complaints; The researchers are required to inform ERC, promptly of any complaints made or expressions of concern are raised, in relation to the project.

Amendments to the approved project (Including changes in personals); requires the submission of a Request for Amendment to ERC and must not begin without written approval from ERC. Substantial variations may require a new application.

Annual Reports; Continued approval of this project is dependent on the submission of an Annual Report.

Extensions of Approval; The researches are required to submit a request to extend the period of validity of approval one month prior to approval expiry date along with the progress/Annual report.

Final Report; A final report should be provided at the conclusion of the project.ERC should be notified if the project is discontinued before the expected date of completion.

Monitoring; Project may be subject to an audit or any other form of monitoring by ERC, at any time

Dr. K. D. P. Wijesinghe
MBBS,MSC,MD,(Med. Admin)(Colombo)
Msc (Health Service Management)(Australia)
Director
Castle Street Hospital for Women
Colombo - 8
SRI LANKA.

Director,
Castle Street Hospital for Women.

Dr. U.D.P. RATNASIRI
[illegible] (UK)
Consul[illegible] & Gynaecologist
Ca[illegible] Hospital for Women
Colombo - 08.

Chairman,
ERC committee, CSHW.

දුරකථන / தொலைபேசி / Telephone 0112696231 / 0112696232 / 0112695529
ෆැක්ස් / தொலைநகல் / Fax 0112695244
විද්‍යුත් තැපැල / மின்னஞ்சல் / email info@cshw.health.gov.lk
වෙබ් අඩවිය / இணையதளம் / Website www.cshw.health.gov.lk
