# Supplementary material for: Perceptions on respectful maternity care in Sri Lanka: Study protocol for a mixed-methods study of patients and providers
Source: PLoS One. 2021 May 5;16(5):e0250920. doi: 10.1371/journal.pone.0250920 (PMC8099093; doi:10.1371/journal.pone.0250920)
Supplement: Attachment — Submitted filename: point by point.docx [file pone.0250920.s006.docx]

**Point by point response.**

Requested changes were made and highlighted in red. We have not submitted or published this article elsewhere and no competing interests. The data which will be gathered during this this study will be available upon a reasonable request from the corresponding author.

My sincere gratitude goes to the editors and reviewers for their generous effort in improving this manuscript. All the comments raised by the editors and reviewers have been addressed point by point.

**Editor’s comments**

1. Please ensure that your manuscript meets PLOS ONE's style requirements, including those for file naming. – Thank you. These changes were made in the manuscript in line 1-22.
2. Please include a copy of the interview guide used in the study, in both the original language and English, as Supporting Information, or include a citation if it has been published previously. – Thank you. These changes were made in the manuscript. S2,3,4 Files have been included in line 579-581.
3. Please include additional information regarding the survey or questionnaire used in the study and ensure that you have provided sufficient details that others could replicate the analyses. For instance, if you developed a questionnaire as part of this study and it is not under a copyright more restrictive than CC-BY, please include a copy, in both the original language and English, as Supporting Information.- – Thank you. These changes were made in the manuscript. S5 Files have been included in line 582. However, Part-4: FOBS was not added [we got it from a Sweden author-reference given] and it will be available upon a reasonable request. Two questions in FOBS has mentioned in the manuscript text [437-440]. WP-RMC tool was under copy right from the original authors (Ayoubi, Pazande et al 2020) and could not be included here.
4. Please state whether you validated the questionnaire prior to testing on study participants. Please provide details regarding the validation group within the methods section. – Thank you. These changes were made in the manuscript and detailed validation procedures mentioned in lines 424 for questionnaire-1, 446-469 for FOBS and WP-RMC in 474-498.
5. We note that you have stated that you will provide repository information for your data at acceptance. Should your manuscript be accepted for publication, we will hold it until you provide the relevant accession numbers or DOIs necessary to access your data. If you wish to make changes to your Data Availability statement, please describe these changes in your cover letter and we will update your Data Availability statement to reflect the information you provide. - The data which will be gathered during this this study will be available upon a reasonable request from the corresponding author, not through the repository.
6. Please include a caption for figure 1. . – Thank you. These changes were made in the manuscript in line 175.
7. Please include captions for your Supporting Information files at the end of your manuscript, and update any in-text citations to match accordingly. Please see our Supporting Information guidelines for more information: <http://journals.plos.org/plosone/s/supporting-information>. . – Thank you. These changes were made in the manuscript in lines 575-584.
8. Additional Editor Comments (if provided):

Please read carefully the feedbacks from the referees and provide a point by point answer. – Thank you. These changes were made in the manuscript and this cover letter.

Please use the appropriate reporting guidelines checklists, such as STROBE and COREQ or SRQR (for qualitative research) Thank you. This was added (SRQR).

Please also specify if the study has been registered in any platform – No, not registered.

Please also specify study timelines – Thank you. These changes were made in the manuscript and a Gantt chart added in lines 532 and 584 and Fig. 2 was also added for Gantt chart.

**Reviewers' comments:**

**Reviewer 1**

1. First, the research questions are not clearly stated on last paragraphs of background section, but on lines 102-104 and 113-115; also, research questions are not in line with the following section: Thank you. These changes were made in the manuscript and refer lines 137-141.

Objectives of the study (line 177). Please revise. Changes were added and in line 164-168. I would suggest the use of reporting guidelines. Checklists, such as STROBE and COREQ or SRQR (for qualitative research). It would help to improve the whole paper comprehension. - Thank you. These changes were made in the manuscript (SRQR was used).

In addition, reference numbering is confusing and not organized in the order that articles were cited in the text (for example, in the first paragraph [lines 78-85] were cited references 8, 11 and 21). I recommend that the authors closely review and edit the paper. -Thank you. These changes were made in the manuscript.

2. Second, on methods would be helpful to provide more information regarding setting and specific hospital units described (example, well-baby clinic). I am assuming that De Soysa Hospital for Women (DSHW) should be also mentioned on lines 467 and 468, please review and edit if necessary. -Thank you. These changes were made in the manuscript. DSHW is also mentioned in lines 186-189.
3. Third, please clarify recruitment procedures. For example, for the qualitative phase: how representativeness of the sample will be monitored?

-Thank you. Changes were made in the manuscript describing the representativeness. Included in lines 245-252.

“Participant recruitment will be done while maintaining a diversity to yield a good representativeness during sample selection. Pregnant and postnatal mothers will be recruited starting from uncomplicated cases, then instrumental delivery, elective/emergency caesarean delivery, awaiting a vaginal birth after caesarean (VABC), not willing for a VBAC in this pregnancy, maternal request for a caesarean, and mothers with a history of previous delivery in a different hospital and/or private sector accordingly.  For HCP, representativeness will be maintained by selecting general labour ward staff to well-experienced ones, staff from units where labor companion is being practiced, and not being practiced at the moment.

During the initial introduction and discussion, if investigators feel that the relevant subject is not appropriate, interviewing will be abandoned and another subject will be selected. If the postnatal women are giving insufficient information while staying the hospital, postnatal interviews will be extended into the postnatal well-baby clinics where mothers are expected to give less socially desirable responses as they are not bound to the labour ward anymore.  Recruiting and interviewing will be continued until the phenomena under the study are well understood and data saturation is reached”.

4. For the quantitative phase, please consider that the sample size was calculated using “100% response rate” and this assumption is not supported by results of similar studies.

-Thank you. These changes were made in the manuscript. Considering a 10% non-response rate, a final sample size of 355 was taken as mentioned in line 380.

In addition, exclusion criteria described for both women and health professionals during qualitative and quantitative phases are different throughout the manuscript (page 9 [lines 198-205] vs page 19 [lines 404-423]). Please revise and closely edit the section accordingly. -Thank you. These changes were made in the manuscript. Inclusion and exclusion criteria were corrected [lines 224-232 and 363-364].

4. Fourth, authors described that "separate interview guides will be prepared for" health professionals and mothers, but the three additional files cited (line 274) were not available for review. -Thank you. These changes were made in the manuscript and all the files attached as supporting information (S2,3,4 Files). See lines 579-580.

Also, intended validation procedures (line 285) are not clearly described. Please provide more details of procedures, including references and additional files. -Thank you. These changes were made in the manuscript under the quantitative phase for FOBS [lines 434-460] and WP-RMC scales [lines 446-469]. Refer the manuscript please for these lines as above.

5. Finally, authors outlined as a limitation that the principal investigator are in the upper-level hierarchy of health professionals but they do not clearly specify if PI currently is working at DSHW and/or CSHW. Considering that all in-depth interviews will be conducted by the PI and the possibility of bias, in particular on health professionals’ results, would be helpful to provide few examples of planned mitigating actions to address this limitation.

-Thank you. These changes were made in the manuscript. Several steps to reduce this effect were added to lines 559-566.

“PI being in the upper-level of hierarchy of HCP in CSHW has to be acknowledged as a limitation. However, a person with knowledge and experience on obstetric procedures is essential in interviewing as the areas indicate specific medical information and knowledge. Effect of PI will be less at DSHW as he is attached to the CSHW. Data collection by additional medical officers and nurses who are not directly involving with labour care in CSHW and DSHW will also minimize the effect of data collection solely by the PI. Since the study will be to conducted in two hospitals, this potential bias may be minimized with different working and interviewing sites of the investigators”.

**Reviewer 2**

1. Nonetheless, although the study design is described in detail in the manuscript, I believe it is appropriate to streamline the background inherent the sub-chapters and the methods description because they look too redundant. -Thank you. These changes were made in the manuscript and streamlined accordingly to paragraphs on importance of quality of maternity care, definition and domains of RMC, implications of RMC, Importance of RMC in the Sri Lankan context and justification, aims of the study [lines 79-169 and methods also streamlined].
2. In this context, I suggest to review the form of the manuscript translating some topics from the "Backgorund" to the "Strengths and limitations" session. -Thank you. These changes were made in the manuscript. Please refer the lines 536-554.

Thank you

Dr.Malitha Patabendige
