# Supplementary material for: Perceptions on respectful maternity care in Sri Lanka: Study protocol for a mixed-methods study of patients and providers
Source: PLoS One. 2021 May 5;16(5):e0250920. doi: 10.1371/journal.pone.0250920 (PMC8099093; doi:10.1371/journal.pone.0250920)
Supplement: Attachment — Submitted filename: PLOS ONE RMC Review 12 2020.docx [file pone.0250920.s007.docx]

**PLOS ONE**

Maternal and healthcare providers’ perceptions on respectful maternity care: study

protocol for a mixed-methods study.

Manuscript Number: PONE-D-20-03776R1

Article Type: Registered Report Protocol

Full Title: Maternal and healthcare providers’ perceptions on respectful maternity care: study

protocol for a mixed-methods study.

Summary and Impression

This is a research protocol report describing a mixed methods study design for analyzing RMC in Sri Lanka. The report lays out in detail the steps needed to understand and replicate the methods the authors are using. The design of the study answers the study question and contributes new knowledge to the maternal health community.

Major Issues

Major issues were identified during the prior revisions.

Minor Issues

Page 4, Line 96-98. The final two sentences of the paragraph do not flow with the material. Unless there is to be a larger discussion of the global caesarean epidemic, I recommend that both of these sentences can be removed.

Page 6, Line 137-141. This paragraph can be removed and if needed, a summative, single addition added to the list in the prior paragraph’s final sentence.

Page 10, Line 223 (and inclusion criteria overall). Are you unnecessarily limiting the scope of your sample by only including “Birth of a live healthy baby.” If RMC influences outcomes for mothers and newborns, including either newborns with complications or who died during/post-delivery may have substantial value. Please explain your rational for this inclusion criteria in the paper.

Page 10, Line 229. Please provide the rational for excluding HCWs with <1 year of obstetric experience. HCWs with less experience in obstetrics may have an informative viewpoint.

Page 11, Line 256 (and other locations in abstract/text). Please explain how you will identify socially desired responses, if responses are being influenced by the location. Limited responses should be relatively straightforward to detect but responses being influenced by the location more challenging.

Page 12, Line 273. Please make explicit what from the previous items that the “This” is that will minimize potential bias.

Page 13, Line 292. Will all data be independently coded by 2 investigators? Later in the manuscript it seems like it will however, this should be clarified here and how any disagreements will be resolved.

Page 13, Line 308-314. The citation is enough, and you can remove the list.

Page 14, Linen 319-320. The first sentence is repetitive with subsection title and not needed.

Page 14, Line 328. Add brief statement on how the interview guides and note-taker forms will be pretested.

Page 14, Line 335-336. #6 does not have detail and therefore does not add value. Suggest removing.

Page 15, Line 356-360. Inclusion criteria for the quantitative phase. Are women not able to read sufficiently, excluded from the quantitative portion as two parts are self administered. If so, please add to Limitations. If not, please explain how this will be handled.

Page 16, Line 377. Typo please correct (Z&)

Page 16, Line 380. Please provide a reference if available to the anticipated 10% non-response rate. This seems low for this vulnerable population.

Page 21, Line 486-. Translation is described repeatedly in the manuscript. This may be better consolidated into a single area in the methods section for brevity.

Page 23, Line 537. Please succinctly state what the research gap is specifically.

Page 24, Line 559. The line “However, proving proper information might reduce this” is unclear.

Page 23-24. Limitations. Is having a male interviewer a potential limitation? Should this be explicitly stated?

Overall, please check carefully for typos and grammar.
