# Supplementary material for: Perceptions on respectful maternity care in Sri Lanka: Study protocol for a mixed-methods study of patients and providers
Source: PLoS One. 2021 May 5;16(5):e0250920. doi: 10.1371/journal.pone.0250920 (PMC8099093; doi:10.1371/journal.pone.0250920)
Supplement: Attachment — Submitted filename: PLOS ONE RMC point by point Review Feb 28 2020.docx [file pone.0250920.s008.docx]

**PLOS ONE**

**Perceptions on respectful maternity care in Sri Lanka: Study protocol for a mixed-methods study of patients and providers**.

Manuscript Number: PONE-D-20-03776R1

Article Type: Registered Report Protocol

My sincere gratitude goes to the editors and reviewers for their generous effort in improving this manuscript. All the comments raised by the editors and reviewers have been addressed point by point.

Thank you

Dr.Malitha Patabendige

**Reviewer #1:**

The current version of the protocol has improved compared with the first one, but, some very important methodological issues were still not appropriately addressed by authors.

Regarding recruitment procedures, ii still not clear how both pregnant or postnatal women would be recruited by clinical characteristics (lines 246 to 250: “uncomplicated cases, instrumental delivery, elective/emergency caesarean delivery, awaiting a vaginal birth after caesarean (VABC), not willing for a VBAC in the current pregnancy, maternal request for a caesarean, and mothers with a history of previous delivery in a different hospital and/or private sector”) without prior access to medical records or privacy violations.

**– Thank you, these controversial issues have been addressed.**

For health care professionals, recruitment procedures described on manuscript are vague: it’s not clear the number of professional categories that would be invited to participate and in which proportion those categories will be present in the final sample. In addition, it’s not clear how the specification “units where labor companion is being practiced and not being practiced at the moment” would influence health workers sample size or composition.

**– Thank you, these controversial issues have been addressed. Details have been included about the HCP and how to deal with units without labour companionship.**

Regarding interviews, it is still not clear how interviewer guides both for pregnant/mothers and health care professionals should be used. The documents provided as supplementary files are poor detailed and difficult to be used in a standardized way without additional instructions.

**– Thank you, these controversial issues have been addressed. I personally contacted Prof.Afulani and went through her interview guides and learnt a lot to improve interviewer guides.**

Also, the criteria (why and how) “interviewing will be abandoned” (lines 254 and 255) or “extended into postnatal well-baby clinics” (line 257) is very subjective and not clear described on manuscript (add a selection bias for complete interviews?).

**– Thank you, these controversial issues have been addressed and removed.**

I also recommend to provide a reference about the “framework approach” that will be used to analyze interviews content (lines 296 and 301) and to provide the checklist file that will be used for quality control of qualitative phase (lines 328-329).

**– Thank you, these controversial issues have been addressed. References provided.**

Regarding assessment tools, the questionnaire 1 (intended to collect “maternal opinion on mode of delivery and childbirth”) should be validated in a sample of mothers. According to authors (lines 423-425) the face validity was done “with the participation of obstetricians, public health specialists”. The proposed use of questionnaire 3 (WP-RMC) “for women who were admitted to the labour suite irrespective of mode of delivery” is not in accordance with the original reference n.30 and it’s not clear how further modifications will be performed (lines 486-487). In addition, in line 409 seems that the publication cited on reference number 30 is about Sri Lanka protocol (“changes accepted by the original authors of WP-RMC in Iran (30)”).

**– Thank you, these controversial issues have been addressed. Pre-testing of questionnaire 01 among a sample of 25 postnatal women has been done.**

The quality of the English throughout the manuscript would be improved by asking a native English-speaking to review the text. The lack of a market copy of the manuscript among the files uploaded by authors have made difficult the review of this version of the manuscript.

**– Thank you, copy with tracked-changes has been attached for your reference.**

**Reviewer #3:**

This manuscript, “Maternal and healthcare providers’ perceptions on respectful maternity care: study protocol for a mixed-methods study,” describes a planned observational study to use interviews with obstetric patients and providers and interview-led obstetric patient survey assessment of the Fear of Birth Scale to improve understanding of respectful obstetric care in Colombo, Sri Lanka.

First and foremost, I commend the study team for designing a study to help address the very important problem of disrespectful obstetric care, which has been overlooked in much of the world, particularly South Asia. However, I think there is substantial room for improvement in the study design and writing of the paper.

Major concerns:
1) Utility of designing a new obstetric care scale for patients, and based off a scale designed in Sweden. Why not use an existing tool, such as this one that has been validated in India and now used prolifically? Afulani PA, et al. Validation of the person-centered maternity care scale in India. Reproductive Health 2018;15(147). Other major papers in this field are missing, such as <https://www.ncbi.nlm.nih.gov/pmc/articles/PMC5764229/> The authors write, “Currently, there are tools have developed to measure the women’s expectations of RMC in global literature.” I think the authors mean no tools, which is untrue. Also the incorrect English of this sentence and throughout the paper really makes it hard to follow, although I am sympathetic to English not being the team’s first language.

**– Thank you, these important issues have been addressed. References have been added. Justification has provided why we prefer Iranian tool- WP-RMC. We have also acknowledged the value of important work by Afulani et al on RMC.**

2) I have serious concerns about the validity of the interviews. On the provider side, very little detail is given and it’s unclear what is expected to be found. The paper cites a prior study of “poor attitudes” of Sri Lankan obstetricians of allowing labor companions, which is a standard component of respectful maternity care. On the patient side, I strongly disagree that the PI, a male authority figure tied to the women’s care, is the appropriate person to lead all patient interviews and I think it will lead to inaccurate interviews with patients. I strongly think they should be led by a female who is not a healthcare provider – ideally a mother and someone of the same ethnicity.

**– Thank you, we agree with your concerns and a female group of data collectors was included. Details added for the providers’ side.**

3) The paper is quite hard to follow – it could be much better organized and I think the reviewer comments could have been more fully addressed.

- **Thank you, the entire manuscript was re-written and completely changed.**

Specific comments:

Title: Is confusing – I thought when I first read it that this study was about obstetric providers’ perceptions of respectful maternity care. Suggested rephrase to “Perceptions on respectful maternity care in Sri Lanka: Study protocol for a mixed-method study of patients and providers.”

- **Thank you, we have changed the title as suggested.**

Introduction: Authors conflate “disrespectful maternity care” and “obstetric violence,” which the introduction largely talks about. The Introduction is long and not well focused (4 pages). I would devote more text to the specific Colombo setting. (Example, why is this study needed in addition to obstetric violence and obstetric patient experience studies that have been done in India and other LMICs?)

- **Sri Lanka is a unique setting among other LMIC, so then Colombo setting has been justified.**

  Qualitative aim: Besides issues mentioned above, I think the study should just interview women postpartum during delivery hospitalization. It does not make sense to interview women hospitalized for delivery who have not yet delivered. HCP is not well defined. I do not agree with only including healthy women with healthy babies – much is to be learned about the experiences of women when complications arise.
- **Thank you, we have included only the vaginally delivered postnatal women. Women who had complications also included. HCP and the aims were defined.**

Unclear why purposive sampling and what is meant by the investigator will “abandon” the interview if he decides the subject is “not appropriate.” This opens up substantial risk of selection bias. Very little detail on the HCP interviews.

- **Thank you, these have been addressed completely. Controversial points *[investigator will “abandon” the interview if he decides the subject is “not appropriate*] have been removed.**

  Quantitative aim: This aim is overall confusing. I think that the FOBS from the Swedish study will be adapted based on the interviews? In the power calculation, it says the main outcome is “fear of birth” but I have no idea who is being compared. I don’t follow the analysis plan – it’s very vague.
- **Thank you, we have removed the FOBS from the protocol and focused only on the RMC. Accordingly, analysis plan was made clear.**

**Reviewer #4: PLOS ONE
Maternal and healthcare providers’ perceptions on respectful maternity care: study protocol for a mixed-methods study.**

Summary and Impression

This is a research protocol report describing a mixed methods study design for analyzing RMC in Sri Lanka. The report lays out in detail the steps needed to understand and replicate the methods the authors are using. The design of the study answers the study question and contributes new knowledge to the maternal health community.

Major Issues

Major issues were identified during the prior revisions.

- **Thank you.**

Minor Issues

Page 4, Line 96-98. The final two sentences of the paragraph do not flow with the material. Unless there is to be a larger discussion of the global caesarean epidemic, I recommend that both of these sentences can be removed.

- **Thank you, we have addressed these issues and the manuscript was updated accordingly.**

Page 6, Line 137-141. This paragraph can be removed and if needed, a summative, single addition added to the list in the prior paragraph’s final sentence.

- **Thank you, we have addressed these issues and the manuscript was updated accordingly.**

Page 10, Line 223 (and inclusion criteria overall). Are you unnecessarily limiting the scope of your sample by only including “Birth of a live healthy baby.” If RMC influences outcomes for mothers and newborns, including either newborns with complications or who died during/post-delivery may have substantial value. Please explain your rational for this inclusion criteria in the paper.

- **Thank you, we have addressed these issues and the manuscript was updated accordingly. Women with complications also added.**

Page 10, Line 229. Please provide the rational for excluding HCWs with <1 year of obstetric experience. HCWs with less experience in obstetrics may have an informative viewpoint.

- **Thank you, we have addressed these issues and the manuscript was updated accordingly. They have also included.**

Page 11, Line 256 (and other locations in abstract/text). Please explain how you will identify socially desired responses, if responses are being influenced by the location. Limited responses should be relatively straightforward to detect but responses being influenced by the location more challenging.

- **Thank you, we have addressed these issues and the manuscript was updated accordingly. This could be a limitation.**

Page 12, Line 273. Please make explicit what from the previous items that the “This” is that will minimize potential bias.

- **Thank you, we have addressed these issues and the manuscript was updated accordingly. Controversial points removed.**

Page 13, Line 292. Will all data be independently coded by 2 investigators? Later in the manuscript it seems like it will however, this should be clarified here and how any disagreements will be resolved.

- **Thank you, we have addressed these issues and the manuscript was updated accordingly.**

Page 13, Line 308-314. The citation is enough, and you can remove the list.

Page 14, Linen 319-320. The first sentence is repetitive with subsection title and not needed.

Page 14, Line 328. Add brief statement on how the interview guides and note-taker forms will be pretested.

- **Thank you, we have addressed these issues and the manuscript was updated accordingly.**

Page 14, Line 335-336. #6 does not have detail and therefore does not add value. Suggest removing.

Page 15, Line 356-360. Inclusion criteria for the quantitative phase. Are women not able to read sufficiently, excluded from the quantitative portion as two parts are self-administered. If so, please add to Limitations. If not, please explain how this will be handled.

- **Thank you, we have addressed these issues and the manuscript was updated accordingly.**

Page 16, Line 377. Typo please correct (Z&)

Page 16, Line 380. Please provide a reference if available to the anticipated 10% non-response rate. This seems low for this vulnerable population.

Page 21, Line 486-. Translation is described repeatedly in the manuscript. This may be better consolidated into a single area in the methods section for brevity.

- **Thank you, we have addressed these issues and the manuscript was updated accordingly.**

Page 23, Line 537. Please succinctly state what the research gap is specifically.

Page 24, Line 559. The line “However, proving proper information might reduce this” is unclear.

Page 23-24. Limitations. Is having a male interviewer a potential limitation? Should this be explicitly stated?. Overall, please check carefully for typos and grammar.

- **Thank you, we have addressed these issues and the manuscript was updated accordingly.**
